# The Relationship between Lifestyle Factors and Body Compositionin Young Adults

**DOI:** 10.3390/ijerph14080893

**Published:** 2017-08-08

**Authors:** Lovro Štefan, Marko Čule, Ivan Milinović, Dora Juranko, Goran Sporiš

**Affiliations:** 1 Faculty of Kinesiology, University of Zagreb, 10 000 Zagreb, Croatia; sporis.79@gmail.com; 2 Faculty of Economics and Business, University of Zagreb, 10 000 Zagreb, Croatia; mcule@efzg.hr (M.Č.); imilinovic@efzg.hr (I.M.); 3 Boutique Fitnes Studio “Vježbaonica”, Center for Recreationand Fitness, 10 000 Zagreb, Croatia; dora.juranko@kif.hr

**Keywords:** university students, body composition, substance use, physical activity, diet

## Abstract

*Background:* Little is known of how lifestyle factors might influence on body composition parameters in young adults from Croatia. The main purpose of the present study was to investigate the relationship between the lifestyle factors and body composition in young adults. *Methods:* In this cross-sectional study, participants were 271 university students (59.0% of women). Body composition was measured by using bioelectric impendance analysis (BIA). Blood pressure and heart rate were measured according to standardized protocol and Mediterranean diet adherence (MD), physical activity (PA) and psychological distress (PD) were assessed with validated questionnaires. *Results:* Self-rated health (SRH) and PA were inversely associated with weight, body-mass index (BMI), fat-mass percentage and blood pressure in men and with weight, BMI, fat-mass percentage and heart rate in women. Higher levels of SRH and PA were positively associated with fat-free mass percentage in both men and women. Smoking was positively associatedwith BMI and fat-mass percentage in women and with heart rate in men. Alcohol consumption was positively associated with weight and BMI in women and fat-mass percentage and heart rate in men, yet inversely associated with fat-free mass percentage only in men. PD was positively associated with weight and blood pressure in men and with BMI, fat-mass percentage and blood pressure in women. *Conclusions:* Our study shows that higher levels of SRH, MD and PA are related with healthy body composition parameters in young adults. Special interventions and policies that enhance PA and MD and decrease substance use and misuse (SUM) and PD should be implemented within the university school systems.

## 1. Introduction

In recent years, obesity has become one of the biggest public health concerns in the world [1]. It represents a well-known cause for premature death [2], coronary heart disease, hypertension, sleeping problems and lower quality of life [3]. As for adult population, most recently, an increasing trend of obesity has affected young adults, especially university students [4]. An increment of body-mass index (BMI) in this specific population can be explained by the fact that most of the university students undergo through lifestyle changes, such as leaving home, going to university [5], leave familiar context and starting work [6] and having increased autonomy in decision-making [7]. This transitional period between childhood and adulthood affect energy balance leading to weight gain [8]. Specifically, the prevalence of obesity in young adults is 2.9–14.3% in China [9], 11.0–37.5% in India [8,10], around 17% in USA [11] and 12.4–31.6% in Latin America and South Africa [12,13].

Since the prevalence of obesity in young adults is relatively high, factors related with body composition parameters, such as fat-mass percentage, fat free-mass percentage, weight and BMI need to be identified, in order to keep prevention programs more effective [14]. It has been documented, that physical activity (PA) is inversely related with BMI, waist circumference (WC), waist to height ratio (WHR) and fat-mass percentage and positively related with fat-free mass percentage [15]. Moreover, in one recent study, higher Mediterranean diet adherence (MD) was inversely related with overweight and obesity status and fat-mass percentage independently of age, socioeconomic status (SES), gender, study center and PA [16]. Another potential factor related with body composition is self-rated health (SRH) [17]. A study by Tang et al. [17] showed that poor SRH was related with increased weight, both underweight and overweight/obese nutritional status and higher fat-mass percentage. On the other hand, smoking and alcohol consumption have negative consequences on body composition parameters, that is, heavy smokers and drinkers (those smoking a greater number of cigarettes/day and drinking more alcohol beverages/day have greater body weight and BMI status [18,19,20,21]. Psychological distress is also one of the potential factors related with body composition. Specifically, it has been reported, that being in the underweight and obese category increasing the odds of medium and high psychological distress in a general population [22].

Most of the aforementioned studies have been conducted on general populations [17,18,19]. As mentioned before, young adulthood is a transitional period, often characterized with poor diet habits, following a diet accompanied by a high consumption of fast food and high-calorie products, which can potentially lead to increased risk of cardiovascular and metabolic diseases [23]. Moreover, 40.0–50.0% of young adults do not meet the recommendations of doing at least 150 min of moderate-to-vigorous PA weekly [24] and only 9.0% of them meet the criterion of 10.000 steps every day [25]. Finally, common stressful events are often accompanied by higher smoking and cigarette consumption, which potentially lead to increased fat-mass percentage [26]. According to the presented evidence, lifestyle factors influence on body composition. However, little is known of how potential lifestyle factors are associated with body composition parameters in young adults, especially in Croatia. Thus, the main purpose of the present study was to investigate the associationbetween the lifestyle factors and body composition in young adults.

## 2. Materials and Methods

### 2.1. Study Participants

In this cross-sectional study, participants were 350 university students randomly selected from one faculty in the city of Zagreb, Croatia. Randomization of participants was done with replacement by drawing unique code for each participant from the box and having equal probability of selection. Fifty participants did not want to participate in the study and 29 of them had missing data. At the end, 271 participants (mean age 19.81 ± 1.26; 59.0% of women) were selected. Prior the study, participants needed to give an oral informed consent to participate in the study. Also, they were told that the study was volountary and they could withdrawn at any time. The data collected in the study were anonymous and in accordance to the Declaration of Helsinki. Institutional Review Board of the leading author approved the study (number 16/2017).

### 2.2. Lifestyle Factors

To assess health status, we used one-item question:“How would you rate your health?“ with five possible answers: (1) very poor; (2) poor; (3) fair; (4) good and (5) excellent [27]. It has been well-documented, that SRH serves as a good predictor of mortality [28]. To assess the MD, we used the Mediterranean Diet Quality Index (KIDMED) questionnaire. The questionnaire is consisted of 16 questions, of which 4 of them denote poor MD (consumption of fast food, baked goods, sweets and skipping breakfast) and 12 of them denote good MD (consumption of oil, fish, fruits, vegetables, cereals, nuts, pulses, pasta or rice, dairy products and yoghurt). Questions denoting poor adherence are scored with −1, while those denoting good adherence are scored with +1. Scores of 16 questions were then summed (0–12 points), with higher score indicating higher level of MD [29]. This questionnaire has been previously used and validated in similar samples of participants [30]. As a measure of PA, we considered students’ total physical activity in the last 7 days. Physical activity was assesses with validated International Physical Activity Questionnaire (IPAQ—short version) [31]. We grouped the participants into three categories: (1) physically inactive; (2) minimally active and (3) HEPA active. Smoking and alcohol consumption were grouped into 4 categories: (1) never; (2) occasional; (3) regular and (4) current regular [17]. We used Kessler questionnaire to assess the level of PD [32]. It consists of 6 questions with 4 possible answers: (1) none of the time; (2) sometimes; (3) often and (4) all of the time. The responses were summed up (range between 0 and 24), where higher score indicated higher level of PD [32].

### 2.3. Body Composition Assessment

To assess body composition characteristics of the participants, we used bioelectric impedance analysis (BIA) (Model TBF-310, Tanita Corporation of America, Inc., Arlington Heights, IL, USA; Tanita-BIA). The whole body composition procedure was done in the morning, after the first urine void and an overnight fast [33]. It has been well-documented, that BIA is correlated with X-ray absorptiometry method and represents a simple and reliable tool for determining body composition [34]. As additional variables, we measured body height to the nearest 0.1 cm, by using a stadiometer (SECA). Body weight was measured to the nearest 0.1 kg by using a calibrated electronic scale. BMI was calculated as weight in kg divided by height in m^2^. Blood pressure and heart rate were measured three times in a sitting position after a 5 min rest period using a standard mercury sphygmomanometer blood pressure cuff according to the American Heart Association’s standardized protocol [35]. Specifically, blood pressure was measured by using a standard mercury sphygmomanometer on the right mid-arm in the same level as the heart. The average systolic blood pressure was taken. Heart rate was measured by putting index and middle finger on the radial artery on the opposite wrist and counting heart beats in 1 min.

### 2.4. Data Analysis

Numerical variables are presented as means ± standard deviations and median with interquartile (IQR) range. Categorical variables are presented as frequencies (N) and percentages (%). Gender differences in numerical variables were analyzed by using Student *t*-test and Man-Whitney U-test, respectively. Differences between categorical variables were analyzed with Chi-square test. The relationship between the lifestyle factors and body composition parameters were calculated with Spearman’s coefficient of correlation. Finally, we performed multiple regression analysis, in order to investigate the association between the set of lifestyle factors and each body composition parameter. We calculated beta (β) coefficients with the coefficient of correlation (R) and coefficient of determination (R^2^), in order to determine the % of variance in the dependent variable (each body composition parameter) which can be explained by the set of independent variables. We performed the multiple regression analysis separately for both men and women and separately between each dependent variable (weight, BMI, fat-mass percentage, fat-free mass percentage, blood pressure and heart rate) and the set of independent variables (SRH, MD, PA, smoking, alcohol consumption and PD). Before we performed multiple regression analysis, we tested regression models for multi-collinearity using the variance inflation factors (VIF); normality of residuals using the normal probability plot and histogram of residuals and heteroscedasticity using the standardized residuals vs. predicted plot. In men, the VIFs were between 1.37 and 1.63 and between 1.13 and 1.53 in women, indicating no multi-collinearity. Durbin Watson test showed no first order linear auto-correlation in both men and women. Other regression assumptions were met for all regression models. Significance was set up at α ≤ 0.05 and it was two-sided. All the analysis done in this study were performed in The Statistical Package for Social Sciences, version 23 (IBM Corp., Armonk, NY, USA).

## 3. Results

Table 1 shows basic descriptive statistics of the study participants. As expected, men are taller and heavier and have higher values of BMI than women. Biologically, women have higher values of fat-mass percentage (31.78% in women vs. 22.29% in men, *p* < 0.001) and heart rate (84.89 bmp in women vs. 79.33 bmp in men, *p* < 0.001), while men have higher values of muscle-mass percentage (*p* < 0.001) and blood pressure (*p* < 0.001). Higher percentage of men are engaged in health-enhanced physical activity (HEPA) category compared with women (37.5% in men vs. 18.2% in women, *p* < 0.001). Interestingly, no significant differences occurred in SRH, MD and PD between genders. Also, there were no significant differences in smoking (*p* = 0.243) and alcohol (*p* = 0.276) consumption between men and women.

The relationship betweenthe lifestyle factors and body composition parameters are presented in Table 2. As shown, SRH, MD and PA are significantly inversely related with weight, BMI, fat-mass percentage and blood pressure in both men and women. Furthermore, SRH and PA are inversely related with heart rate in both genders while MD is significantly inversely related with heart rate only in the sample of women. All of the aforementioned variables are positively related with muscle-mass percentage in both men and women. Significant positive relations are found between smoking and weight, BMI and heart rate in both genders, while postitive relations between smoking and fat-mass percentage as well as blood pressure were found only in women. Also, significant inverse relations between smoking an muscle-mass percentage are found only in women. Alcohol consumption and PD aresignificanlty postitively related with weight, BMI andfat-mass percentage, and negatively related with muscle-mass percentage in both genders. Furthermore, alcohol consumption is significantly positively related with heart rate on both samples while postitive relation between alcohol and blood pressure is signicicant only in a sample of women. PD is also significantly postively related with blood pressure (men and women) while positive relation between PD and heart rate is found only in woman.

Table 3 shows the association between the lifestyle factors and body composition parameters. SRH was inversely associated with weight, BMI, fat-mass percentage, blood pressure and heart rate in both men and women. MD was positively associated with fat-free mass percentage only in men (β-0.17) and inversely associated with blood pressure in both men and women and with heart rate only in women (β-0.16). PA was positively asssociated with fat-free mass percentage in both genders, yet inversely associated with weight, BMI and fat-mass percentage in both men and women, but only with blood pressure in women (β-0.20) and heart rate in men (β-0.18). Smoking was positively associated with BMI and fat-mass percentage only in women and with heart rate in men. Alcohol consumption was positively associated with weight and BMI in women and with fat-mass percentage and heart rate in men. Finally, PD was positively associated with weight and blood pressure in men and with BMI, fat-mass percentage and blood pressure in women.

## 4. Discussion

The main purpose of the present study was to investigate the relationship between the lifestyle factors and body composition in young adults.

Our findings showed, that SRH was inversely associatedwith weight, BMI, fat-mass percentage, blood pressure and heart rate in both men and women. It has been reported, that increased weight, BMI, WC and body fat percentage influence on functional capacity and mental status [17]. Moreover, weight and BMI are shown to be a better indicators than WC and WHR, pointing out that general adiposity might be a better indicator than central adiposity of SRH [17]. We also found that self-rated health was positively associated with muscle-mass percentage in both genders. Such results are in line with other findings [36,37]. Specifically, both physical and cardiorespiratory fitness have been related with good SRH [36,37]. It has also been reported in a similar population, that adolescents who report having poor SRH are less fit than their fit peers [38]. SRH was also inversely related with blood pressure in men and heart rate in both men and women. One previous study has shown, that participants with blood pressure and heart rate within the referent values are more likely to report good self-rated health [39].

Next, our results showed that MD was positivelyassociated with fat-free mass percentage only in men and inversely associated with blood pressure in both men and women and with heart rate only in women Similar relationship has been shown in adult population [40] and children and adolescents [41,42]. Components of the MD, such as fruits, vegetables and olive oil, are mainly responsible for the protection against hypertension and cardiovascular diseases. Moreover, the high consumption of products rich with minerals and plant foods rich with antioxidants contribute to the health of vascular system and reduction of high arterial blood pressure [43]. Most recently, a study conducted on a large sample of European adolescents showed, that participants, who were overweight and obese had increased odds of reporting low MD [44]. We also found that MD is inversely related with heart rate, which is consistent with some previous findings [45]. MD is rich with omega-3 fatty acids, which can potentially reduce heart rate and suppress the automaticity of cardiac contraction [45].

PA was positively asssociated with fat-free mass percentage in both genders, yet inversely associated with weight, BMI and fat-mass percentage in both men and women, but only with blood pressure in women and heart rate in men. Our results are similar with some previous findings [15,46,47]. Specifically, Zaccagni et al. [15] showed, that physically active women had higher values of fat-free mass percentage compared to the less active individuals, while men showed a lower fat-mass percentage and BMI. Both Lohman et al. [46] and Tudor-Locke et al. [47] reported inverse relationship between fat-mass percentage and different levels of accelerometry and pedometer-determined PA. Also, PA has been shown to lower both blood pressure and heart rate values [48]. However, the mechanism by which PA may reduce blood pressure is still unclear. It has been reported, that aerobic exercise has beneficial effects on insulin sensitivity and autonomic nervous system functioning [49], while resistance training effects on vasoconstriction regulation [50].

In our study, smoking was positively associated with BMI and fat-mass percentage only in women and with heart rate in menAlcohol consumption was positively associated with weight and BMI in women and with fat-mass percentage in men. Previous findings have shown, that among smoker, an increased amount of smoking tends to be positively related with BMI in men, while waist-to-hip ratio (WHR) is positively related with amount of cigarettes smoked per day in both men and women [18,19]. In adolescents, smoking at least 10 cigarettes per day increases the risk of adult abdominal obesity [51]. Smoking causes an acute increase in both blood pressure and heart rate, due to a nicotine act as an adrenergic agonist, which may release vasopressin [52]. Similar to smoking, alcohol consumption increases BMI, WHR, WC and fat-mass percentage [20,21]. Alcohol serves as a suppressor for fat oxidation and can increase fat sysnthesis [53]. Physiologically, ethanol affects the transcription of genes involved in muscle hyperthrophy, where inhibits testosteron and increases cortisol levels, an opposite trend in muscle hyperthrophy [54]. Participants who reported consuming alcohol more often had higher values of both blood pressure. In one meta-analysis, where the alcohol reduction was an intervention between the active and control group, showed that alcoholic beverage reduction led to lower values of both systolic and diastolic blood pressure [55]. Finally, our findings showed, that alcohol consumption was positively related with heart rate only in men. Ryan & Lowes [56] reported the same results, where alcohol consumption was a significant positive predictor of 24 hour heart rate.

Finally, PD was positively associated with weight and blood pressure in men and with BMI, fat-mass percentage and blood pressure in women. Previous findings have reported, that stress level, often characterized by negative life events and problems, is related with overall and central adiposity in children [57]. As highlighted before, being in the underweight and obese category increases the odds of medium and high psychological distress in a general population [22]. From the physiological point of view, changes in metabolism, i.e.,increased visceral fat, is often caused by a dysregulation of the stress system and the production of stress hormones, such as cortisol [58]. Also, PD has been shown to increase the odds of hypertension, due to a poor mental hygiene and often consumption of cigarettes and alcoholic beverages [59].

Our study has several limitations. First, due to a cross-sectional design, we cannot exclude the possibility of reverse causality. Second, to assess adherence to the MD and PA, we used previously validated questionnaires. However, the usage of subjective measures often leads to overestimation [60]. Finally, we conducted a study on a small sample (*N* = 272) of university students. Future studies should take into account students from other faculties with different professions and lifestyle habits.

## 5. Conclusions

The main purpose of this study was to investigate the relationship between the lifestyle factors and body composition in young adults.

We found strong and inverse association between the SRH and PA with weight, BMI, fat-mass percentage, blood pressure and heart rate and strong positive association with muscle-mass percentage. Smoking, alcohol consumption and PD was positively associatedwith BMI andfat-mass percentage, and inversely associatedwith muscle-mass percentage.

Special interventions and policies that enhance PA and MD and decrease substance use and misuse (SUM) and PD should be implemented within the university school systems.

## Figures and Tables

**Table 1:** Basic descriptive statistics of the study participants.

Study Variables	Total Sample (*N* = 271)	Men (*N* = 112)	Women (*N* = 159)	*p*-Value
	mean ± s.d.	mean ± s.d.	mean ± s.d.	
**Numerical variables**				
Age (years)	19.81 ± 1.26	19.88 ± 1.33	19.75 ± 1.20	0.703
Height (m)	1.73 ± 0.09	1.81 ± 0.07	1.68 ± 0.06	<0.001
Weight (kg)	70.23 ± 15.01	81.89 ± 14.21	62.02 ± 8.90	<0.001
BMI (kg/m^2^)	23.19 ± 3.59	24.83 ± 3.82	22.03 ± 2.92	<0.001
Fat-mass (%)	27.86 ± 7.90	22.29 ± 6.73	31.78 ± 6.11	<0.001
Fat-free mass (%) **	32.12 ± 6.07	38.08 ± 4.29	27.92 ± 2.68	<0.001
Blood pressure (mm/Hg) **	121.74 ± 16.91	131.03 ± 16.05	115.19 ± 14.25	<0.001
Heart rate (beats per minute) **	82.59 ± 14.74	79.33 ± 15.44	84.89 ± 13.82	<0.001
SRH (points) **	4 (3–5) *	4 (3–5) *	4 (3–5) *	0.747
MD (points) **	4 (2–8) *	3 (2–8) *	4 (2–8) *	0.114
PD (points) **	5 (3–13) *	6 (3–13) *	5 (3–13) *	0.092
**Categorical variables**	N (%)	N (%)	N (%)	
Smoking ***				
Never	116 (42.8)	46 (41.1)	70 (44.0)	
Occasionally	46 (17.0)	25 (22.3)	21 (13.2)	
Often	24 (8.9)	10 (8.9)	14 (8.8)	
Very often	85 (31.4)	31 (27.7)	54 (34.0)	0.243
Alcohol consumption ***				
Never	66 (24.4)	29 (25.9)	37 (23.3)	
Occasionally	157 (57.9)	58 (51.8)	99 (62.3)	
Often	43 (15.9)	22 (19.6)	21 (13.2)	
Very often	5 (1.8)	21 (13.2)	2 (1.3)	0.276
PA ***				
Physicallyinactive	131 (48.3)	49 (43.8)	82 (51.6)	
Minimally active	69 (25.5)	21 (18.8)	48 (30.2)	
HEPA active	71 (26.2)	42 (37.5)	29 (18.2)	<0.001

No asterisk denotes using Student *t*-test for indepent samples; * denotes using median and interquartile range (IQR); ** denotes using Man-Whitney U-test; *** denotes using Chi-square test; HEPA = health-enhanced physical activity; BMI = body-mass index; SRH = self-rated health; MD = Mediterranean diet adherence; PA = physical activity; PD = psychological distress; *p* < 0.05.

**Table 2:** The relationship between the lifestyle factors and body composition parameters, stratified by gender.

Study Variables	Weight	BMI	Fat-Mass Percentage	Fat-Free Mass Percentage	Blood Pressure	Heart Rate
	r Coefficient	r Coefficient	r Coefficient	r Coefficient	r Coefficient	r Coefficient
**SRH**						
Men	−0.47 ***	−0.45 ***	−0.46 ***	0.47 ***	−0.31 ***	−0.30 ***
Women	−0.33 ***	−0.41 ***	−0.40 ***	0.38 ***	−0.16 *	−0.31 ***
**MD**						
Men	−0.26 **	−0.29 **	−0.28 **	0.24 **	−0.26 **	−0.09
Women	−0.19 *	−0.21 **	−0.17 *	0.17 *	−0.32 ***	−0.24 **
**PA**						
Men	−0.28 **	−0.29 **	−0.28 **	0.26 **	−0.28 **	−0.20 *
Women	−0.30 ***	−0.37 ***	−0.36 ***	0.35 ***	−0.31 ***	−0.20 *
**Smoking**						
Men	0.31 ***	0.23 *	0.12	−0.16	0.06	0.31 ***
Women	0.19 *	0.29 ***	0.23**	−0.23 **	0.18 *	0.20 *
**Alcohol consumption**						
Men	0.35 ***	0.34 ***	0.41 ***	−0.39 ***	0.09	0.29 **
Women	0.29 ***	0.37 ***	0.31 ***	−0.23 **	0.21 **	0.21 **
**PD**						
Men	0.33 ***	0.33 ***	0.25 **	−0.23 *	0.30 ***	0.15
Women	0.19 *	0.24 **	0.26 ***	−0.27 ***	0.32 ***	0.20 *

BMI = body-mass index; SRH = self-rated health; MD = Mediterranean diet adherence; PA = physical activity; PD = psychological distress; * *p* < 0.05; ** *p* < 0.01; *** *p* < 0.001.

**Table 3:** Multiple regression analysis between the lifestyle factors and body composition parameters, stratified by gender.

Study Variables	Weight	BMI	Fat-Mass Percentage	Fat-Free Mass Percentage	Blood Pressure	Heart Rate
	β Coefficient	β Coefficient	β Coefficient	β Coefficient	β Coefficient	β Coefficient
**SRH**						
Men	−0.31 ***	−0.39 ***	−0.42 ***	0.33 ***	−0.37 ***	−0.22 *
Women	−0.29 ***	−0.34 ***	−0.32 ***	0.28 ***	−0.06	−0.25 *
**MD**						
Men	−0.06	−0.05	−0.04	0.17 *	−0.18 *	0.05
Women	−0.05	−0.04	−0.05	−0.02	−0.18 *	−0.16 *
**PA**						
Men	−0.19 *	−0.17 *	−0.19 *	0.23 **	−0.09	−0.18 *
Women	−0.21 **	−0.21 **	−0.22 **	0.16 *	−0.20 **	−0.11
**Smoking**						
Men	0.11	0.01	0.01	0.08	−0.08	0.17 *
Women	−0.04	0.20 *	0.21 *	0.14	0.05	−0.01
**Alcohol consumption**						
Men	0.08	0.06	0.25 *	−0.17 *	−0.21 *	0.17 *
Women	0.19 *	0.18 *	0.13	−0.02	0.09	0.01
**PD**						
Men	0.17 *	0.13	0.06	−0.07	0.24 **	0.01
Women	0.06	0.13 *	0.13 *	−0.16 *	0.19 *	0.06
**R coefficient**						
Men	0.62 ***	0.58 ***	0.64 ***	0.64 ***	0.50 ***	0.43 ***
Women	0.53 ***	0.63 ***	0.56 ***	0.48 ***	0.49 ***	0.40 ***
**R^2^ coefficient**						
Men	0.38 ***	0.34 ***	0.41 ***	0.41 ***	0.25 ***	0.19 ***
Women	0.28 ***	0.40 ***	0.31 ***	0.23 ***	0.24 ***	0.16 ***

BMI = body-mass index; SRH = self-rated health; MD = Mediterranean diet adherence; PA = physical activity; PD = psychological distress; R = coefficient of correlation; R^2^ = coefficient of detrmination; * *p* < 0.05; ** *p* < 0.01; *** *p* < 0.001.

## Abbreviations

BIA	bioelectric impedance analysis
BMI	body-mass index
BPM	beats per minute
HEPA	health-enhanced physical activity
MD	mediterranean diet adherence
PA	physical activity
PD	psychological distress
SES	socioeconomic status
SRH	self-rated health
SUM	substance use and misuse
WC	waist circumference
WHR	waist-to-hip ratio
